# 149. Increasing Oxacillin Resistance of S*taphylococcus lugdunensis* Over Time, Utilizing Whole Genome Sequencing to Characterize Resistance

**DOI:** 10.1093/ofid/ofac492.227

**Published:** 2022-12-15

**Authors:** Kristin Constance, Yuriko Fukuta, Elizabeth Penner, Todd M Lasco, Audrey Wanger, Blake M Hanson, Masayuki Nigo

**Affiliations:** University of Texas Health Science Center, Houston, Texas; Baylor College of Medicine, Houston, Texas; UT Health Houston McGovern Medical School, Houston, Texas; Baylor St. Luke's Medical Center, Houston, Texas; McGovern Medical School, Houston, Texas; University of Texas Health Science Center, Houston, Texas; UT Health Mc Govern Medical School, Houston, Texas

## Abstract

**Background:**

*Staphylococcus lugdunensis (SL)* is a coagulase negative staphylococci (CoNS) demonstrating more virulent pathogenicity compared to other CoNS. With implementation of rapid diagnostics such as multiplex PCR on blood culture and use of MALDI-TOF (MALDI), CoNS can now be routinely specifically identified as *SL*. We sought to describe the antibiotic susceptibility of *SL* over time, and to utilize whole genome sequencing (WGS) to identify the characteristics of oxacillin (OXA) resistant *SL*.

**Methods:**

Retrospective review of all culture isolates positive for *SL* from two major hospital systems, Memorial Hermann Hospital System (MHHS) and Baylor St. Lukes Medical Center (BSLMC) in Houston, TX. MALDI was implemented in 2016 at BSLMC and 2019 at MHHS. MHHS utilizes Microscan®, and BSLMC utilizes Vitek2® for susceptibility testing. For this study, all duplicated isolates within a 2-week period were excluded. Six patient isolates, three OXA resistant and three susceptible, were sent for WGS and analysis.

**Results:**

Between 2014 and 2021, 744 culture isolates were identified as *SL,* 325 from MHHS and 419 from BSLMC (Fig 1). An increasing trend was observed at MHHS over time, however this trend was not observed at BSLMC. 83.6% (622/744) of isolates were susceptible to OXA. An overall trend towards increasing resistance was observed over time (Fig 2). Six isolates, three OXA susceptible (S) and three resistant (R), were sent for WGS. The R isolates were found to share the same sequence type (ST3), and while they were not clonal, were closely related and all harbored an SCCmec cassette most closely related to SCCmec type IVg.

**Figure:**
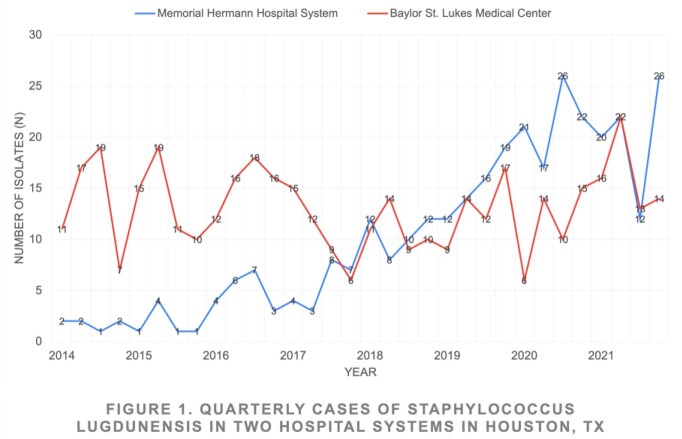

**Figure:**
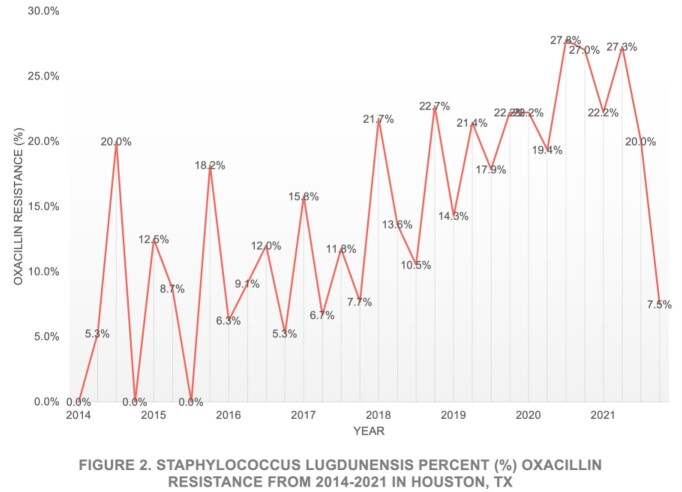

**Conclusion:**

In our study, an increasing number of isolates were identified at MHHS over time. This finding may be related to implementation of MALDI. Overall OXA susceptibility was lower than expected at 83.6%, when compared to a prior large-scale United States based study in 2017 demonstrating 95.3% susceptibility. This finding of developing resistance is concerning. While WGS analysis of R isolates did not demonstrate clonality, it did show close relation. This data may suggest expansion of an emerging lineage, however more isolates will need to be studied for conclusion given limited sample size.

**Disclosures:**

**All Authors**: No reported disclosures.

